# SENP7 inhibits glioblastoma metastasis and invasion by dissociating SUMO2/3 binding to specific target proteins

**DOI:** 10.1515/med-2024-1052

**Published:** 2024-10-07

**Authors:** Jixing Zhang, Hongshan Zheng, Peng Liang

**Affiliations:** Harbin Medical University Cancer Hospital, Harbin, China; Tianjin Huanhu Hospital, Tianjin, China

**Keywords:** GBM, SENP7, infiltrative, MMP9

## Abstract

**Background:**

The poor surgical efficacy and recurrence of glioblastoma (GBM) are due to its lack of visible infiltrative features. Our bioinformatics study suggests that low expression of small ubiquitin-like modifier (SUMO)-specific protease 7 (SENP7) indicates poor prognosis in GBM.

**Objectives:**

This study investigated the effect of SENP7 expression on the invasion, migration, and proliferation of GBM cells and aims to identify the SUMO target proteins affected by SENP7.

**Methods:**

SENP7 expression was analyzed in eight GBM tumor samples and four GBM cell lines, comparing them to normal brain tissue. The effect of SENP7 overexpression on GBM LN229 cell migration, invasion, and proliferation was examined through *in vitro* assays. Furthermore, four SUMO target proteins involved in tumor invasion and proliferation (CDK6, matrix metalloproteinase-9 [MMP9], AKT, and HIF-1α) were studied to explore SENP7’s molecular mechanism.

**Results:**

SENP7 expression was significantly lower in GBM tumors compared to normal tissue. SENP7 overexpression in LN229 cells inhibited migration and invasion without affecting proliferation. Overexpression reduced the levels of MMP9, AKT, and HIF-1α, but not CDK6. Immunohistochemical analysis showed decreased MMP9 and CD31 levels, suggesting reduced tumor invasion and angiogenesis. However, SENP7 overexpression did not affect tumor growth *in vivo*.

**Conclusions:**

SENP7 inhibits GBM invasion by dissociating proteins associated with tumor invasion from SUMO2/3, providing a potential target for future GBM therapies.

## 1 Introduction

Glioblastoma (GBM) is the most malignant glioma among astrocytomas [1]. Growing throughout the supratentorial cerebral hemisphere, tumors are primarily seen beneath the cortex [2]. Deep structures and many brain lobes are frequently invaded by it, exhibiting infiltrative growth. Through the corpus callosum, it can potentially spread to the opposing hemisphere [3]. Tumor infiltration destroys brain tissue, causing a series of focal symptoms, with patients experiencing varying degrees of hemiplegia, hemisensory impairment, aphasia, and hemianopsia [4]. Due to the rapid growth of tumors, 70–80% of patients have a disease course of 3–6 months, and only 10% have a disease course of more than 1 year [5]. However, what makes neurosurgeons struggle is that the invasive growth characteristics of GBM make it almost impossible for them to remove all tumor cells [6]. Therefore, it is imperative to search for new drugs that target GBM invasion.

Protein SUMOylation, a dynamic and reversible post-translational modification form of proteins that is widely present in a variety of organisms, is controlled by the small ubiquitin-like modifier (SUMO) family, which is mostly composed of SUMO1, SUMO2, and SUMO3 [7]. This intricate regulation mechanism involves the cooperative actions of several different enzyme systems and cofactors. These enzyme systems comprise SUMO deactivating enzymes, E2 binding enzymes, and E1 activating enzymes, which are in charge of SUMO dissociation, binding, and activation, respectively [8]. By covalently attaching to the lysine residues of substrate proteins, SUMOylation controls the structure and functionality of substrate proteins [9]. Its characteristics include: (1) a target protein can undergo SUMOylation modification on different lysine residues. (2) The same protein can be modified by multiple SUMO proteins simultaneously, forming multiple SUMOylation modifications, further increasing the complexity and diversity of modifications. (3) SUMO modification can interact and regulate with other modification methods, such as ubiquitination and phosphorylation, to form a modification network [10].

SUMO-specific protease (SENP) specifically deSUMOylation modifies from substrate proteins and works together with SUMOs to regulate the SUMOylation state of substrate proteins, and then regulates cell function [11]. SENP1, SENP2, SENP3, SENP5, SENP6, and SENP7 are members of the SENP family; their intracellular localization and substrate specificity vary [12]. SUMO1, SUMO2, and SUMO3 can generally be dissociated from their target proteins by SENP1 and SENP2. Additionally, SUMO2 and SUMO3 can specifically be dissociated from target proteins by SENP3 and SENP5. Furthermore, SUMO2 and SUMO3 polymers are primarily dissociated from target proteins by SENP6 and SENP7. [13,14,15]. Because of their high specificity and low side effects when compared to medications targeting the SUMO family [13,14], pharmaceuticals targeting members of the SENP family are therefore well-suited for the development of precision medicine.

The present study employed bioinformatics research to ascertain whether a correlation exists between the decreased expression of SENP6 and SENP7 and a poor prognosis in GBM. This prompted us to investigate the role of SENP6 and SENP7 in GBM further. However, due to the unavailability of a commercially available antibody for SENP6, this investigation exclusively examined the impact of restoring SENP7 expression on tumor cell proliferation and invasion in the SENP7-deficient GBM cell line LN229. Our findings indicate that overexpression of SENP7 can reduce tumor cell invasion but has no effect on GBM proliferation. This discovery offers promising avenues for targeted treatment of highly invasive GBM.

## 2 Materials and methods

### 2.1 Data sources and analysis

Using the UCSC Xena database’s Survival data (*n* = 626), we combined the mRNA expression data of deSUMOylation genes (SENP1, SENP2, SENP3, SENP5, and SENP6) and SUMOylation genes (SUMO1, SUMO2, SUMO3, and UBC9) with the corresponding clinical survival data for 33 cancer types to perform an expression survival analysis. Through the use of the R package “survival,” we apply a univariate COX risk proportional regression model to compare the progression-free survival (PFS) or overall survival (OS) between the low- and high-risk groups.

### 2.2 Human tissue samples

Eight GBM patients who had surgical resection at the Tianjin Fifth Central Hospital (Tianjin, China) between January 2009 and December 2022 provided fresh surgical specimens (tumor and matched neighboring non-tumor tissues). A top pathologist performed the first diagnosis on all frozen samples. To validate the preliminary diagnosis, additional pathologists reexamined paraffin-embedded sections. All tumors have the same characteristics in this group, according to the 5th edition (2021) of the WHO classification of CNS tumors. Detailed information on the patients is shown in Table 1.

**Table 1:** Basic information of eight patients

No.	Sex	Age	Diagnosis	WHO grade	Brain region	Primary/recurrent	Side	GBO success
1	M	72	GBM	4	RT	Primary	R	Yes
2	F	65	GBM	4	RT	Primary	R	Yes
3	F	67	GBM	4	LF	Primary	L	Yes
4	F	73	GBM	4	RF	Primary	R	Yes
5	M	57	GBM	4	RP	Primary	R	Yes
6	F	74	GBM	4	LF-T	Primary	L	Yes
7	M	61	GBM	4	RT-P	Primary	R	Yes
8	F	68	GBM	4	LF	Primary	L	Yes

### 2.3 Lines of cells and cultured cells

The American Type Culture Collection (Maryland, USA) provided the four GBM cell lines A172, SNB19, U251, and LN229 and an immortalized normal human astrocyte cell line HA1800. All of these cell lines were verified to be free of mycoplasma infection. The four GBM cells were grown in Dulbecco’s modified Eagle’s medium (DMEM) supplemented with 10% fetal bovine serum (FBS), 100 U/ml penicillin, and 100 µg/ml streptomycin (all from Gibco; Thermo Fisher Scientific, Inc., Waltham, MA, USA). The HA1800 astrocyte cells were grown in a specialized glial cell culture medium (Zhongqiao Xinzhou Biotechnology Co., Ltd, Shanghai, China), at 37°C in a humidified environment with 5% CO_2_.

### 2.4 Gene transduction

After being subcloned into pCMV-Myc, the SENP7 gene was cloned into pCDH-CMV-MCS-EF1-copGFP, a lentiviral vector. The control group consisted of untreated cells, while the nonsense group consisted of cells transduced with an empty vector. Transduction was carried out in compliance with the manufacturer’s instructions. Briefly, 5 µl of viral suspension (108 titers) was applied to cell monolayers after LN229 cells had been cultivated to 60–70% confluence. Following a 6-h incubation period at 37°C and 5% CO_2_, the viral suspension was withdrawn from the flasks and replaced with new media. With western blotting, the impact of gene transduction was confirmed.

### 2.5 Western blotting

RIPA buffer (Solarbio, Beijing, China) was used to extract proteins from either freshly harvested tumor tissues or cultured cells. The BCA Protein Quantification Kit (Thermo Fisher Scientific, Inc.) was used to measure the quantities of proteins. After that, total proteins (100 µg per lane) were separated on 10% sodium dodecyl sulfate-polyacrylamide gel electrophoresis gels and then put on membranes made of polyvinylidene fluoride (EMD Millipore, Inc.). After that, antibodies specific to target proteins were used to probe the membranes. Table 2 displays details about each antibody. The National Institutes of Health, Bethesda, MD, USA, used ImageJ version 1.48 as the image analysis software to examine the data.

**Table 2:** Details of all relevant antibodies in this study

Antibody	SENP7	CDK6	MMP9	AKT	HIF-1α	GAPDH
Company	Abcam	Invitrogen	Abcam	CST	Abcam	Abcam
Code	ab58422	PA5-27978	ab76003	#9272	Ab1	Ab8245
Dilution	1:800	1:1,000	1:2,000	1:1,000	1:500	1:2,000

### 2.6 Assay for cell proliferation

Using the Cell Counting Kit-8 (CCK-8; Yeasen, Shanghai, China), cell proliferation was assessed. 96-Well plates were used to cultivate the cells in accordance with the experimental groups. After that, we carried out a daily vitality test. 10 µl of CCK-8 (5 mg/ml) was added to each well, and the cells were incubated for 1 h before the absorbance at 450 nm was measured using a microplate reader (Bio-Rad, Hercules, CA, USA) to assess changes in cell proliferation. Ultimately, a standard curve’s cell number was obtained using absorbance data.

### 2.7 Plate clone formation assay

After using 0.25% trypsin to break down each group of cells during the logarithmic growth phase and blowing them into individual cells, the cells are suspended in DMEM culture media containing 10% FBS. After inoculating, grow the cells for 2–3 weeks at a density of 100 cells per dish. After that, fix the cells for 15 min with 4% paraformaldehyde and 20 min with GIEMSA staining. Under a microscope, counted clones, took pictures, and computed the rate at which clones form. (Number of clones/number of inoculated cells) × 100% is the clone production rate.

### 2.8 Healing wounds assay

LN229 cells were seeded into six-well plates, either with or without SENP7 gene transduction, and cultivated until a cell monolayer was formed. With a 200 µl pipette tip, small incisions were then produced in the monolayer cells. Digital pictures of the wounds were taken at 0 and 48 h after scratching using an inverted microscope (Olympus, Japan), and the wound width was calculated using ImageJ. Cell migration was then measured using wound width values.

### 2.9 Assay for cell invasion

To assess cell invasion, 1 × 10^4^ cells were planted into the upper well of transwell chambers, while the lower well was filled with media containing 10% FBS. Transwell chamber membranes were coated with BD Biosciences’ Matrigel for 30 min at 37°C before cells were seeded. Next, the cells were cultured for 48 h. The membranes were stained with crystal violet for 10 min after the medium was taken out of the top chamber. After that, an inverted microscope was used to examine the inserts, and ImageJ was used to count the cells that had entered through the Matrigel.

### 2.10 Cell cycle analysis

After 48 h of culture, LN229 cells, either transduced with or without the SENP7 gene, were frozen in 70% ethanol for 30 min at 4°C. They were then treated for 30 min at 37°C in the dark with RNase (1 mg/ml) and the DNA-binding dye propidium iodide (50 µg/ml). Lastly, a peak fluorescence gate was utilized to distinguish between aggregates during the analysis of red fluorescence using a FacsCalibur™ flow cytometer and CellQuest software version 4.0 (both from BD Biosciences, Franklin Lakes, NJ, USA), all following a standard methodology.

### 2.11 Experimental mouse model

Twenty-four female nude mice weighing between 14 and 16 g and aged 4 weeks were bought from Sibeifu Biotechnology Co., Ltd., in Beijing, China, and kept in the Tianjin Fifth Central Hospital’s Experimental Animal Center. The mice were kept in an environment with regulated temperature (22–24°C) and consistent humidity (40–60%), with a 12-h light/dark cycle and unlimited access to food and drink. Eight naked mice per group were subcutaneously injected with either LN229 cells transduced with or without the SENP7 gene (5 × 10^6^ in 0.2 ml phosphate-buffered solution [PBS]). For a period of 25 days, the growth of the tumor was measured every 5 days using a caliper. Tumor volume (*V*) was computed as follows: *V* = *L* × *W* ^2^ × 0.5, where *L* stands for length and *W* for width. Following the mice’s sacrifice, the tumor weight was ascertained. For the purpose of immunohistochemical studies, the tumor blocks were subsequently embedded in paraffin.

### 2.12 Immunohistochemistry assay

Sections of tissue were blocked in PBS (pH 7.5) containing 5% bovine serum albumin and subsequently treated for an entire night at 4°C with primary antibodies (Abcam) against Ki67 (1:500), CD31 (1:500), and matrix metalloproteinase-9 (MMP9) (1:1,000). Using a universal two-step detection kit (PV-8000, Zhongshan Goldenbridge, Beijing, China), immunoreactivity was found.

### 2.13 Statistical analysis

At least three separate experiments were conducted to obtain all of the experimental data. With the use of GraphPad Prism 6 software (San Diego, CA), all data were analyzed and are shown as means ± standard deviations (SD). One-way analysis of variance or Student’s *t*-test, followed by Tukey’s test, was used to establish statistical significance. For statistical significance, a value of *P* < 0.05 was declared.


**Ethical approval:** The study was authorized by the Tianjin Fifth Central Hospital’s Biomedical Research Ethics Committee.
**Informed consent:** Every patient gave their informed consent.

## 3 Results

### 3.1 The expression level of SENP7 protein in GBM is generally reduced

**Figure 1:**
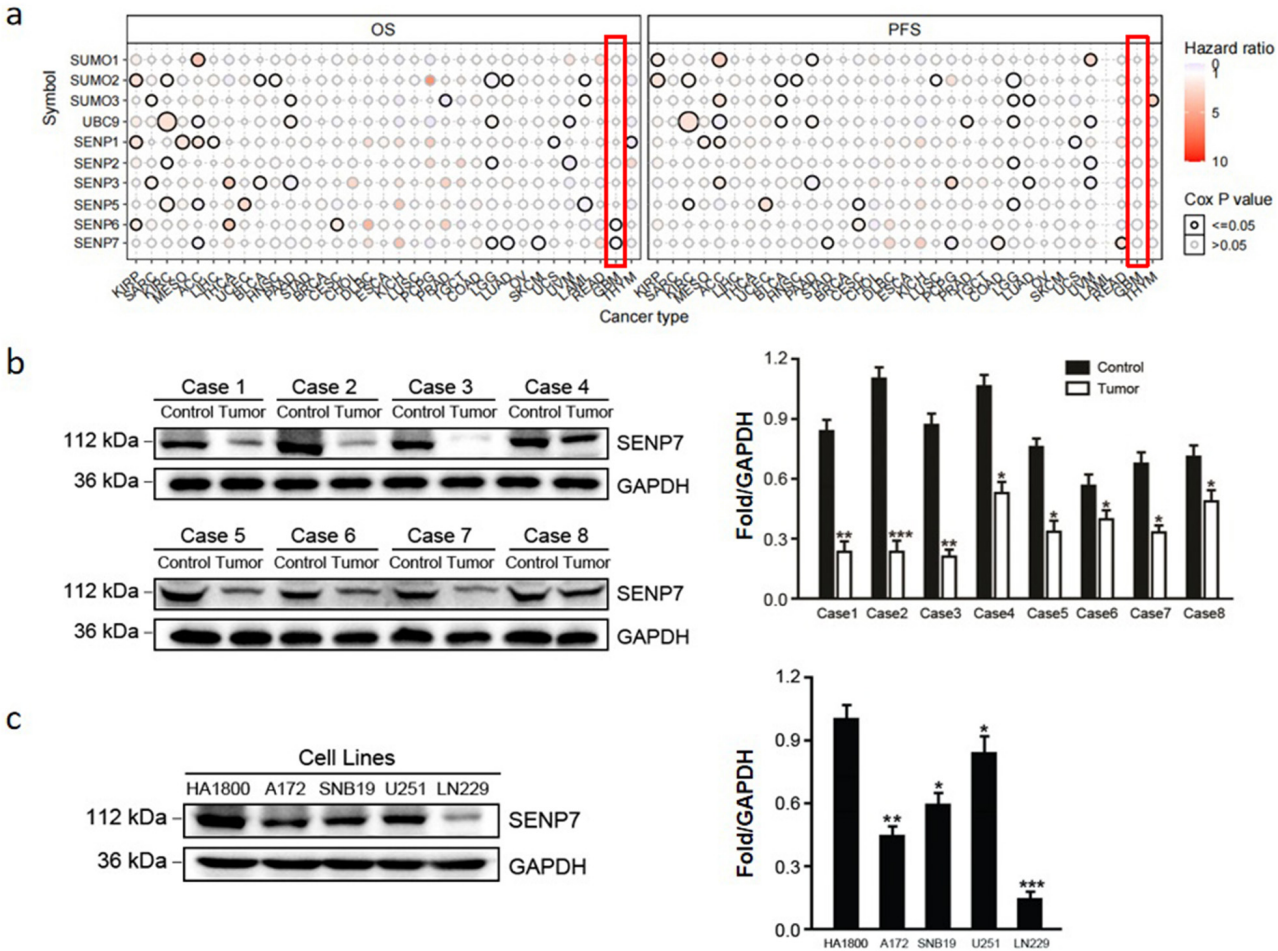
Low expression of SENP7 suggests poor prognosis in GBM patients. SENP6 and SENP7 have specificity characteristics in GBM. SENP6 and SENP7 low expression is associated with a poor OS in GBM patients. The PFS was unaffected by them, nevertheless (a). According to Western blot analysis, GBM tissue and nearby tissue samples expressed the SENP7 protein. KIRP: kidney renal papillary cell carcinoma; SARC: sarcom; KIRC: kidney renal clear cell carcinoma; MESO: mesothelioma; ACC: adrenocortical carcinoma; LIHC: liver hepatocellular carcinoma; THCA: thyroid carcinoma; UCEC: uterine corpus endometrial carcinoma; BLCA: bladder urothelial carcinoma; HNSC: head and neck squamous cell carcinoma; PAAD: pancreatic adenocarcinoma; STAD: stomach adenocarcinoma; BRCA: breast invasive carcinoma; CESC: cervical squamous cell carcinoma and endocervical adenocarcinoma; CHOL: cholangiocarcinoma; DLBC: lymphoid neoplasm diffuse large B-cell lymphoma; ESCA: esophageal carcinoma; KICH: kidney chromophobe; LUSC: lung squamous cell carcinoma; PCPG: pheochromocytoma and paraganglioma; PRAD: prostate adenocarcinoma; TGCT: testicular germ cell tumors; COAD: colon adenocarcinoma; LGG: brain lower grade glioma; LUAD: lung adenocarcinoma; OV: ovarian serous cystadenocarcinoma; SKCM: skin cutaneous melanoma; UCS: uterine carcinosarcoma; UVM: uveal melanoma; LAML: acute myeloid leukemia; READ: rectum adenocarcinoma; GBM: glioblastoma multiforme; THYM: thymoma. (b) As demonstrated by Western blot analysis, SENP7 protein was expressed in one normal glial cell line (HA1800) and four GBM cell lines (A172, SNB19, U251, and LN229). (c) GAPDH served as a reference point for protein expression. The data are displayed as means ± SD (*n* = 3); when compared to the control (Student’s *t*-test), the values are *P < 0.05, **P < 0.01, and ***P < 0.001. OS, PFS, glyceraldehyde-3-phosphate dehydrogenase (GAPDH), and SUMO-specific protease 7 (SENP7).

The baseline characteristics of the eight patients are shown in Table 1, including the information on patients’ age, gender, diagnosis, WHO grade, and other relevant details. In this study, we found that SENP6 and SENP7 have significant specificity characteristics in GBM among all SENP families. The higher expression levels of SENP6 and SENP7 indicate a longer OS for GBM patients, and vice versa (Figure 1a). However, they did not affect the PFS (Figure 1a).

As of right now, not much is known about SENP6 because antibodies necessary for accurate measurement of SENP6 protein expression levels are not available. Consequently, the study’s only focus is on SENP7. First, we identified the SENP7 protein expression level in tumor and surrounding tissue samples from eight individuals with GBM. The findings demonstrated that, in comparison to the surrounding tissues, the expression level of SENP7 protein was significantly lower in all eight observed GBMs (Figure 1b). Subsequently, we identified the SENP7 protein expression levels in one normal glial cell line (HA1800) and four GBM cell lines (A172, SNB19, U251, and LN229). The findings demonstrated that all GBM cell lines, particularly the LN229 cell line, expressed less SENP7 protein than normal glial cells (Figure 1c).

### 3.2 SENP7 mainly affects the invasion and metastasis of GBM, rather than proliferation

**Figure 2:**
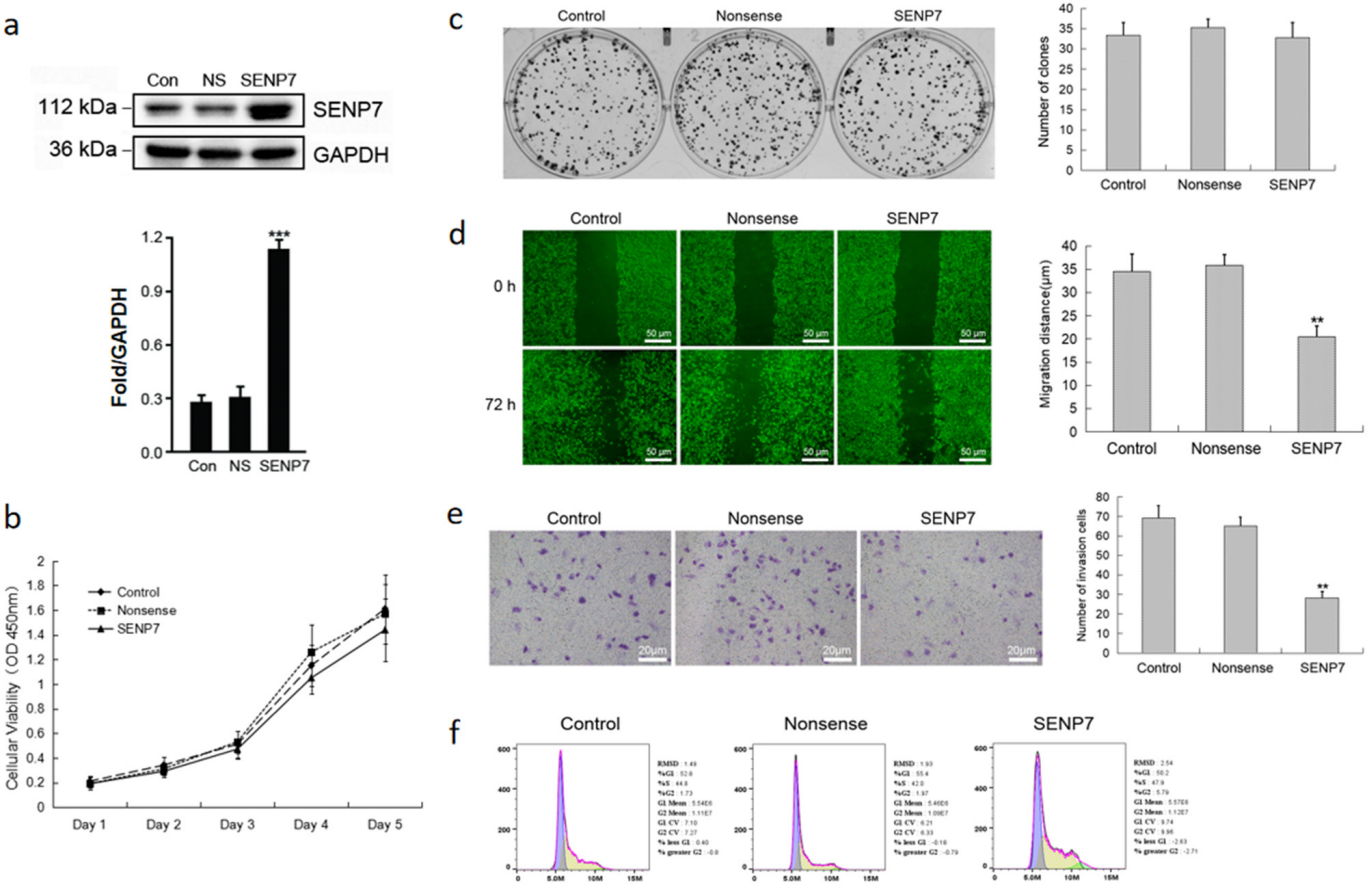
Rather than promoting GBM cell proliferation, SENP7 overexpression blocks GBM cell invasion and migration. SENP7 was successfully transfected in the LN229 cell line, according to Western blot data. (a) The proliferation of LN229 cells transfected with and without the SENP7 gene was demonstrated by the CCK8 experiment. (b) The plate cloning experiment demonstrated that LN229 cells transfected with and without the SENP7 gene were capable of being cloned. (c) LN229 cells transfected with and without the SENP7 gene migrated as demonstrated by the wound healing assay. (d) The Transwell experiment demonstrated the ability of LN229 cells transfected with and without the SENP7 gene to invade. (e) The cell cycle of LN229 cells transfected with and without SENP7 gene transfection was observed using flow cytometry. (f) GAPDH was used as a standard for protein expression. The means ± SD (*n* = 3) are displayed together with **P < 0.01 and ***P < 0.001 for comparisons with the control (Student’s *t*-test). glyceraldehyde-3-phosphate dehydrogenase (GAPDH), and SUMO-specific protease 7 (SENP7).

To further verify whether SENP7 plays a role as a tumor suppressor gene in GBM, using lentivirus vectors, we stably transfected the exogenous SENP7 gene into LN229 cells (Figure 2a) and investigated the impact of SENP7 overexpression on GBM cell migration, invasion, and proliferation. Our findings demonstrated that SENP7 overexpression had no discernible impact on GBM cell growth (Figure 2b). The ability of tumor cells to proliferate and have stem cell potential can be somewhat reflected in their capacity to create cell clones. According to this work, the rate at which GBM cells clone appears to be unaffected by SENP7 overexpression (Figure 2c). Next, we investigated how SENP7 transfection affected GBM cell migration and invasion. The migration (Figure 2d) and invasion (Figure 2e) of GBM cells were dramatically suppressed by overexpressing SENP7, according to the outcomes of scratch and Transwell studies. At last, we also looked at how SENP7 transfection affected the GBM cells’ cell cycle. The cell cycle of GBM was not significantly changed by SENP7 overexpression, according to flow cytometry studies (Figure 2f). The influence of SENP7 on the invasion and metastasis of GBM cells, which in turn impacts the OS of patients, may be the primary source of its anti-tumor action on GBM, according to these studies.

### 3.3 SENP7 inhibits GBM metastasis by affecting multiple SUMO2/3 target proteins

**Figure 3:**
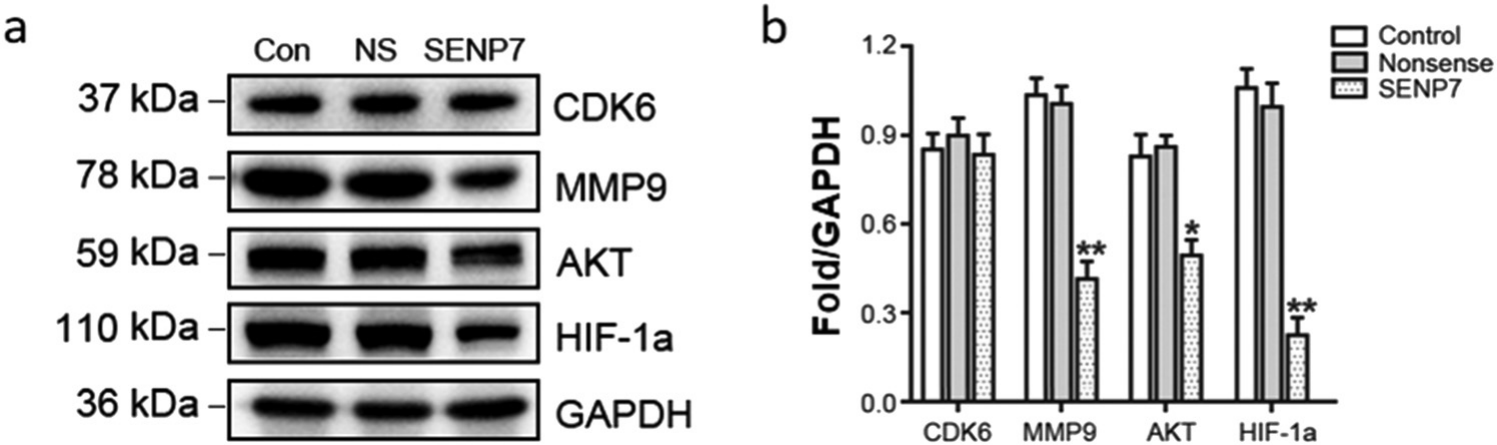
The impact of overexpressing SENP7 on SUMO target protein expression. The effect of SENP7 gene transfection on the expression of SUMO target proteins was demonstrated by the western blot data. (a) The columnar representation originates from. (b) GAPDH was used as a standard for protein expression. The data are shown as means ± SD (*n* = 3); when compared to the control (Student’s *t*-test), the values are *P < 0.05 and **P < 0.01, respectively. SUMO-specific protease 7 (SENP7), glyceraldehyde-3-phosphate dehydrogenase (GAPDH), CDK6 (cyclin-dependent kinase 6) MMP9 (matrix metalloproteinase-9), AKT Protein Kinase B, and HIF-1α (hypoxia-inducible factor 1α).

We identified four verified SUMO2/3 target proteins, which have been shown to have variable effects on the proliferation and metastasis of GBM, to further explore the anti-tumor mechanism of SENP7 on GBM. The outcomes demonstrated that while overexpression of SENP7 significantly decreased the levels of AKT and HIF-1a protein, which are both related to tumor proliferation, migration, and cycle, and MMP9 protein, which is closely related to tumor metastasis, it had no effect on the levels of cell cycle-related protein cyclin-dependent kinase 6 (CDK6) (Figure 3a and b). These findings additionally show that the SENP7 anti-tumor mechanism on GBM is mostly derived from its effect on tumor metastasis, not tumor proliferation.

### 3.4 SENP7 inhibits GBM metastasis and angiogenesis in tumor-bearing mice

**Figure 4:**
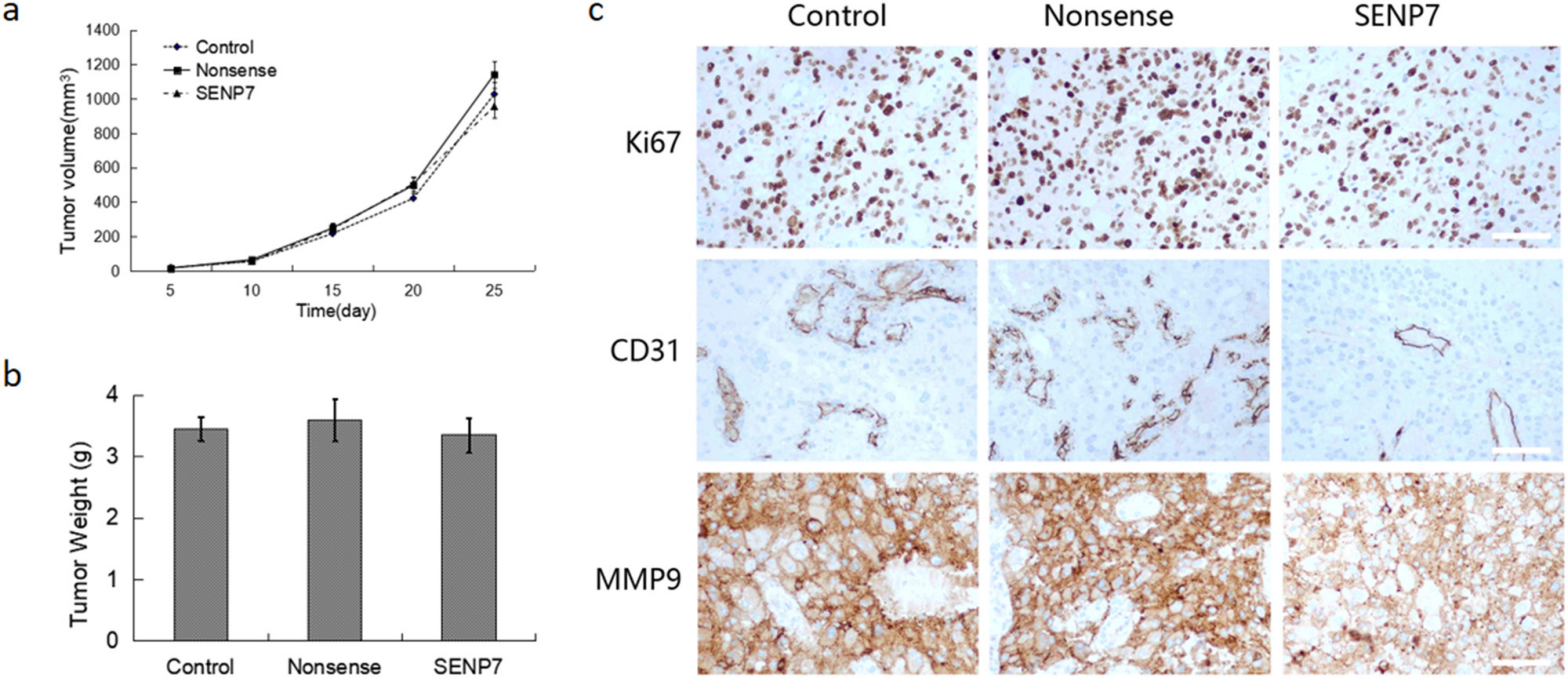
The effect of SENP7 gene transfection on subcutaneous tumors in tumor-bearing mice. The dynamic changes of subcutaneous tumor volume over time in tumor-bearing mice. (a) Compare the weight of subcutaneous tumors after sacrificing tumor-bearing mice. (b) The results of immunohistochemistry experiments showed the expression of related proteins. (c) Data are shown as means ± SD. SUMO-specific protease 7 (SENP7), Platelet endothelial cell adhesion molecule-1 (CD31), Matrix metalloproteinase-9 (MMP9).

A tumor xenograft model was created by subcutaneously injecting LN229 cells, either with or without SENP7 gene transduction, into immunodeficient mice to confirm the tumor-suppressive effects of SENP7 *in vivo*. Tumor volume and weight among the three groups did not significantly differ, as Figure 4a and b demonstrate. We used immunohistochemical detection of tumor tissue to look more closely at the *in vivo* consequences of SENP7 overexpression on GBM. According to the findings, tumor invasion-related protein MMP9 and angiogenic protein CD31 had considerably lower protein levels following SENP7 gene transfection, whereas tumor proliferation indicator Ki67 exhibited virtually no change (Figure 4c). These *in vivo* results further indicate that SENP7 may primarily inhibit the expression of proteins related to tumor invasion and angiogenesis, thereby exerting a tumor-inhibitory effect.

## 4 Discussion

GBM is the most common and fatal glioma in adults, characterized by rapid proliferation, strong invasiveness, and poor clinical prognosis [16]. The molecular complexity, extensive invasiveness, and recurrent tendency of GBM present a challenge for oncologists [17]. Numerous dysregulated genomic pathways and significant interactions with epigenetic modifications are seen in glioma cells [18]. With its potential as a biomarker for tumor categorization, prognosis, and treatment targeting, epigenetic alteration has emerged as a significant player in GBM research [19]. A major regulatory factor for gene expression and functional execution, protein SUMOylation is controlled by members of the SUMO family and is essential for the development and progression of tumors as well as malignant transformation [20]. It is feasible to retrieve SUMOs from target proteins due to the existence of SUMO-specific proteases [21].

According to earlier research, the SUMOylation pathway, which includes SUMOylated products and components of E1 (SAE1) and E2 (Ubc9), is frequently increased in GBM [22,23]. There is no denying the clinical importance and therapeutic benefit of targeted SUMOylation control. Nonetheless, there are not many recognized or efficient SUMO modification inhibitors in use today. Topotecan’s potential use as an adjuvant therapy in a specific group of GBM patients has been highlighted by Bernstock et al.’s demonstration of how it inhibits the SUMOylation of CDK6 and HIF-1α, affecting the cell cycle progression and metabolism of GBM patients [24].

In this study, to do an expression survival study, we first merged the clinical survival data for 33 different cancer types with the mRNA expression data of the SUMOylation and deSUMOylation genes. The findings demonstrated a strong favorable correlation between the OS of GBM and two deSUMOylation enzymes, SENP6 and SENP7, which selectively depolymerize SUMO2/3 polychains, which greatly stimulated our interest. If this specific characteristic is proven to be trustworthy, it will undoubtedly provide important value for the precise treatment of GBM. However, due to our limited understanding of SENP6 and the lack of research tools, this study only focuses on SENP7.

We examined the SENP7 protein expression in eight GBM tissue samples and four GBM cell lines to validate this theory. Based on the data, it was determined that SENP7 protein expression in GBM was substantially lower than in neighboring cancer tissues and normal glial cell lines. This further suggests the potential of SENP7 to play a role as a tumor suppressor gene in GBM. Next, based on *in vitro* SENP7 overexpression experiments and *in vivo* tumor-bearing mouse models, it is suggested that SENP7 inhibitory effect on GBM is mainly reflected in its limitation of tumor infiltration, rather than tumor proliferation.

To further investigate the mechanism by which SENP7 restricts GBM infiltration, we investigated four reported target proteins of SUMOs, which have been confirmed to be associated with GBM invasion and proliferation. CDK6 is an important cell cycle regulatory enzyme [25]. According to Bellail et al., CDK6 is changed by SUMO1 in GBM, and CDK6 SUMOylation stabilizes the protein and promotes the cell cycle, which is necessary for the growth and spread of cancer. During the G1/S transition, CDK6 drives the cell cycle while staying SUMOylated. Additionally, ubiquitin-mediated CDK6 degradation is inhibited by CDK6SUMOylation at Lys 216, which also limits its ubiquitination at Lys 147 [26]. Notably, SUMO1 conjugates CDK6, whereas SUMO2/3 do not [26]. This study found that overexpression of SENP7 hardly affects GBM cell cycle progression, which may be due to SENP7-specific dissociation of SUMO2/3 polymers rather than SUMO1.

MMP9 is a member of the matrix metalloproteinase family, and its primary job is to break down and reorganize the extracellular matrix’s dynamic balance [27]. On the link between SENP7 and GBM, there is not any pertinent research as of now. Our research revealed that MMP9 protein expression was markedly suppressed by SENP7 overexpression. However, as this study did not delve into the molecular mechanism, we cannot arbitrarily assume that SENP7 is directly involved in the regulation of MMP9. Anyway, such results can partially explain the reason why SENP7 inhibits GBM invasion.

Antiviral immunity, proliferation, survival, migration, and metabolism of tumors are all significantly regulated by the phosphatidylinositol-3-kinase (PI3K)/AKT pathway [28]. First documented by Li et al., AKT1 is SUMOylated at many locations, with K276 serving as the primary acceptor site. It can be changed by SUMO1, SUMO2, and SUMO3. Moreover, AKT1 E17K-mediated cell migration, proliferation, and carcinogenesis were all significantly inhibited by SUMOylation loss [29]. AKT1 is the substrate of SENP3, according to Xiao et al.’s discovery that AKT1 hyper-phosphorylation and activation after SENP3 depletion is caused by increased AKT1 SUMOylation [30]. The complicated control of the PI3K/AKT pathway by SUMOylation was reviewed by Vidal et al. [31], who also noted the mechanism’s possible involvement in human disease. Our investigation revealed that SENP7 transfection can lower AKT expression, which could account for some of the protein’s ability to suppress GBM invasion.

HIF-1α is widely present in human cells, even under normoxic conditions [32], but because HIF-1α can only be expressed stably in hypoxic environments, the synthetic HIF-1α protein is quickly broken down by intracellular oxygen-dependent ubiquitin proteases [33]. It has been discovered that around 100 target genes of HIF-1α are connected to the development of inflammation, tumor growth, and hypoxia adaption [34]. The stability of oxygen-sensitive HIF-1α is regulated by post-translational changes that include acetylation, ubiquitination, and hydroxylation [35,36]. According to Bae et al., SUMO-1 modification at Lys(391)/Lys(477) residues increased the expression of HIF-1ɑ, which may stabilize HIF-1ɑ and improve its transcriptional activity [37]. According to this study, SENP7 transgenic mice can lower HIF-1α protein levels. Additionally, overexpression of SENP7 has been shown *in vivo* experiments to decrease angiogenesis in tumor lesions. These findings imply that SENP7 may disrupt angiogenesis by inducing HIF-1̞α deSUMOylation and preventing the activation of its downstream VEGF pathway.

## 5 Conclusion

In summary, the above results make us even more convinced that SENP7 plays a role as a tumor suppressor gene in GBM, and this inhibitory effect mainly comes from its limiting action on tumor invasiveness rather than proliferation. However, based on the complex network regulatory mechanisms in the body and the multi-target characteristics of SUMO, we still cannot confirm the exact mechanism of SENP7 as a tumor suppressor gene. In any case, this study provides important references for drug screening targeting SUMOylation and precise treatment of GBM patients.
